# A systematic review of the clinical survival of zirconia implants

**DOI:** 10.1007/s00784-016-1853-9

**Published:** 2016-05-24

**Authors:** Dena Hashim, Norbert Cionca, Delphine S. Courvoisier, Andrea Mombelli

**Affiliations:** 1 School of Dental Medicine, Division of Periodontology, University of Geneva, Rue Barthelemy-Menn 19, CH-1205 Geneva, Switzerland; 2 University Hospitals of Geneva, Rue Gabrielle-Perret-Gentil 4, CH-1205 Genève, Switzerland

**Keywords:** Zirconia, Dental implants, Systematic review, Survival

## Abstract

**Objectives:**

The aim of this review was to evaluate the clinical success and survival rates of zirconia ceramic implants after at least 1 year of function and to assess if there is sufficient evidence to justify using them as alternatives to titanium implants.

**Materials and methods:**

An electronic search in MEDLINE, EMBASE, and the Cochrane Central Register of Controlled Clinical Trials (CENTRAL) databases was performed in April 2015 by two independent examiners to retrieve clinical studies focusing on the survival rate of zirconia implants after at least 1 year of function. Implant survival was estimated using the overall proportion reported in the studies with a Clopper-Pearson 95 % confidence interval (random effect model with a Der-Simonian Laird estimate).

**Results:**

Fourteen articles were selected out of the 1519 titles initially screened. The overall survival rate of zirconia one- and two-piece implants was calculated at 92 % (95 % CI 87–95) after 1 year of function. The survival of implants at 1 year for the selected studies revealed considerable heterogeneity.

**Conclusions:**

In spite of the unavailability of sufficient long-term evidence to justify using zirconia oral implants, zirconia ceramics could potentially be the alternative to titanium for a non-metallic implant solution. However, further clinical studies are required to establish long-term results, and to determine the risk of technical and biological complications. Additional randomized controlled clinical trials examining two-piece zirconia implant systems are also required to assess their survival and success rates in comparison with titanium as well as one-piece zirconia implants.

**Clinical relevance:**

Zirconia implants provide a potential alternative to titanium ones. However, clinicians must be aware of the lack of knowledge regarding long-term outcomes and specific reasons for failure.

## Introduction

In a world with increasingly heightened esthetic demand, ceramics have become progressively more popular in the dental industry. Nowadays, they are widely used as veneers and abutments for both tooth- and implant-supported all-ceramic restorations, as well as for fabrication of oral implants. Densely sintered alumina (Al_2_O_3_) and yttria-stabilized tetragonal zirconia polycrestal ceramics (Y-TZP) are currently the materials of choice for ceramic abutments [1]. Yet, when it comes to oral implants, zirconia has repeatedly been proven superior to other ceramics in terms of bending strength and fracture toughness [2]. Its low modulus of elasticity and thermal conductivity, low affinity to plaque, and high biocompatibility, in addition to its white color, have made zirconia ceramics a very attractive alternative to titanium in implant dentistry [3–6]. Still, when it comes to disadvantages, low-temperature degradation, also known as ageing, is considered one of zirconia’s major drawbacks. It is a process which results in degradation of the mechanical properties due to the progressive spontaneous transformation of the metastable tetragonal phase into a monoclinic one at temperatures above 200 °C in the presence of water vapor. This causes reduction in the strength, toughness and density of the material. However, reduction in grain size and/or increase in the concentration of stabilizing oxides reduce the transformation rate [7]. An additional concern when using zirconia oral implants has been addressed in an in vitro study evaluating fracture strength. The authors established that both preparation and cyclic loading of zirconia implants can reduce their fracture strength resistance. Nevertheless, they reported that even implants with low mean fracture strength can withstand extended intervals of average occlusal loading [8]. In spite of such limitations, animal studies have repeatedly proven zirconia implants to be comparable, if not superior, to titanium implants in terms of biocompatibility and osseointegration [4, 9–14]. A systematic review [12], evaluating the osseointegration and success of zirconia implants in animal studies, revealed a mean bone-to-implant contact (BIC) greater than 60 % in most of the included studies. One even indicated better bone healing on zirconia cones when compared to titanium [15]. Regardless of such auspicious results, the authors could not recommend the use of zirconia dental implants due to the lack of long term clinical results. Another systematic review [2], which included both animal and clinical studies on alumina and zirconia implants, concluded that there was no difference in the rate of osseointegration between the different implant materials in animal studies. Even though alumina implants were not considered a viable alternative to titanium, zirconia, on the other hand, was viewed as a potential successful implant material despite the lack of supporting clinical data. Ever since, multiple studies evaluating the clinical use of zirconia implants have been published. Yet, different studies examined a variety of implant systems with great diversity in implant design, surface modification, surgical and loading protocols, follow-up period, and prosthetic reconstruction. Furthermore, clinical investigations often used variable definitions for implant success with different clinical indexes. Finally, owing to the increasing number of commercially available ceramic implant systems, as well as the increasing demand for non-metallic and highly esthetic restorations, the clinical performance of zirconia implants has become of substantial interest to the dental practitioner. Hence, the aim of this review was to evaluate the clinical success and survival rates of zirconia ceramic implants after at least 1 year of function, and to assess if there is sufficient evidence to justify using them as alternatives to titanium implants.

## Materials and methods

The method used in this systematic review was adapted from the Preferred Reporting Items for Systematic Reviews and Meta-Analyses (PRISMA) guidelines [16] as well as the recommendations previously established by Needleman [17].

### The focused question

The aim of this review was to answer the following focused questions:

1. What are the clinical survival rates of zirconia ceramic implants?
2. Is there sufficient clinical data on zirconia implants to justify using them as alternatives to titanium implants?

### Search strategy

An electronic search in MEDLINE, EMBASE, and the Cochrane Central Register of Controlled Clinical Trials (CENTRAL) databases was performed for clinical studies published in the English language. No publication year limit was applied so that the search could include the first available year until the first of April 2015. The following search terms (MeSH terms) were utilized: “dental implants” AND (“zirconium oxide” OR “yttria-stabilized tetragonal zirconia polycrystals ceramic”), “dental implants” AND (“zirconia, AND “clinical study”), “dental implants” AND (“zirconium oxide”), “zirconia implants” AND (“clinical” NOT “abutments”), “zirconia implants” AND (“human study” AND “survival rate”), as well as “zirconia implants” AND (“clinical study” AND “failure rate”).

### Inclusion criteria

Publications were considered for inclusion if the following criteria were met:

- Studies reported in the English language in dental journals.
- Clinical studies including at least five human subjects with ceramic implant-supported reconstructions.
- All types of zirconia implants including one- and/or two-piece systems.
- Number of implants specified.
- Observation period of at least 1 year after functional loading.
- Survival and/or success rates clearly stated.
- Clear description of the prosthetic reconstruction.

### Exclusion criteria

Studies not meeting all inclusion criteria were excluded from the review. Publications based on charts, questionnaires, or interviews were also not considered. Due to the limited number of available studies, no further exclusion criteria were specified.

### Selection of studies

Titles and abstracts derived from the search were independently screened by two authors (DH and NC), based on the listed criteria. Full-text articles were then obtained for all titles agreed upon, and disagreements were resolved by discussion. Cohen’s kappa was used to measure inter-reviewer agreement.

### Quality assessment

Assessment of the methodological quality of the included studies was done by the two reviewers (DH and NC). The studies where assessed according to their design, extent of clinical and radiographic examinations, adjustment for potential confounding variables and different surgical protocols, completeness of follow-up, and statistical analysis. Industry funding was also taken into consideration. In light of the mentioned criteria, studies were evaluated as having low, moderate, or high risk of bias [2, 18].

### Data extraction

Data was extracted on each study’s design, publication year, follow-up period, number of patients and implants, implant design and surface characteristics, surgical protocols, survival and/or success rates, details on marginal bone loss (MBL) and prosthetic rehabilitation, as well as failure and complication rates. Any disagreement regarding data extraction was resolved with discussion. If only failure rates were reported, survival rates were calculated after requesting permission from the authors. When data were not clear, the corresponding author was contacted for clarification.

### Statistical analysis

Statistical heterogeneity, assessed using chi-square test and *I*^2^ statistics, was used to estimate the proportion of variance due to heterogeneity among studies. The prevalence of survival of implants was estimated using the overall proportion reported in the studies with a Clopper-Pearson 95 % confidence interval (random effect model with a Der-Simonian Laird estimate). Forest plots were used to show the prevalence estimated in each study with its confidence interval and the weight given to each study in the meta-analyses, along with the overall pooled prevalence.

## Results

**Fig. 1:**
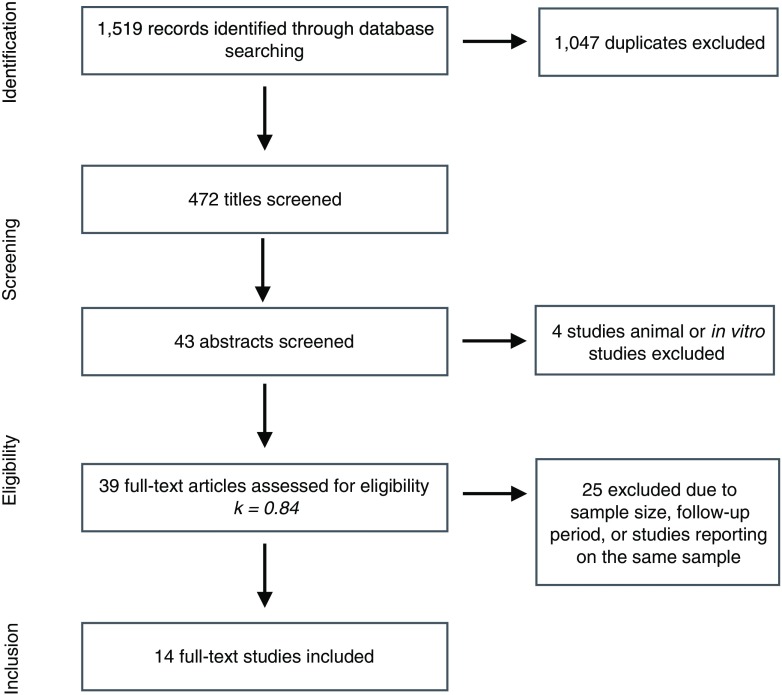
The flow chart for the search strategy

The initial electronic database search yielded 1,519 titles which were independently screened resulting in the consideration of 43 publications. Abstracts were then reviewed and four in vitro or animal studies were further excluded. The remaining 39 studies were reviewed in details resulting in the exclusion of 10 articles which were examining the same groups of patients already included in other publications. This was established after email communication with the authors. Both reviewers agreed on the classification of 36 of the 39 studies, with an estimated kappa of 0.84. In case of multiple papers evaluating the same patient group, the latest or the most relevant publications were selected, with the exception of Spies et al. [19]. This study evaluated the same group of patients examined in two consecutive publications [20, 21]. In spite of being more recent, the publication of Spies et al. was excluded because it focused on the survival of the prosthetic superstructures that were fabricated using a novel hand-layering technique. Sixteen studies were further excluded due to insufficient sample size or short follow-up period. Finally, 14 clinical trials were selected for inclusion in the current review (Fig. 1). Eleven publications examined one-piece implant systems, two evaluated two-piece systems, and one included both one- and two-piece implants. The studies showed variability in implant surface treatment, surgical and loading protocols, prosthetic rehabilitation, and observation period. Hence, meta-analysis was limited to 1 year of functional loading using a random effect model. Only three publications were randomized clinical trials (RCT), whereas the remaining 11 studies were case series with varying designs. Detailed data for the 14 included studies are listed in Table 1.

**Table 1:** Detailed data of the included studies

	Author, year	Study type	Observation period	No. of patients	No. of implants	Implant design	Implant system and surface characteristics	Time and technique of implant placement	Type of prosthetic reconstruction and healing time	Survival rate (%)	Success rate (%)	Mean MBL (mm)
1	Blaschke and Volz 2006	Prospective	2–5 years	34	66	One-piece	Z-Lock 3, VOLZIRKON 1 & 2 (Z-Systems AG, Constance, Germany) CAD/CAM Bio-HIP A zirconia, sandblasted intraosseous section and polished transgingival/abutment portion	NR	Implants protected during the healing phase by splints or prosthesis, then SC after: mand: 4 months max: 6 months	98 % good osseointegration after 1–2 years	NR	NR
2	Pirker and Kocher 2009	Prospective	Mean 18 months	18	18, Group A: 6 group B: 12	One-piece	Single-root analogue zirconia implants group A: sandblasted group B: additional macroretention in interdental space and reduced diameter BL	1–8 days post-extraction by tapping	Immediate limited functional loading composite SC after 3–13 months	Group A: zero survival in 2 months group B: 92	NR	NR
3	Oliva 2010	Prospective	Mean 40.8 months	378	831, uncoated (UC) 249 coated (C) 249 acid etched (ICE) 333	One-piece	Ceraroot zirconia implants (oral iceberg) with 3 different roughened surfaces UC, C, ICE	Immediate, flapless, regeneration, sinus lifts, 1 and 2 stage, or late implant placement, screwed or tapped-in implants	Vacuum stent or immediate provisionally cemented restoration for esthetic areas CAD/CAM final restoration after 4–11 months	NR	Overall 94.9 UC 92.77 C 93.57 ICE 97.6	NR
4	Cannizzaro 2010	RCT	12 months	40	40, occlusal (occ) 20 non-occlusal (non-occ) 20	One-piece	Z-Look 3 zirconia implants (Z-Systems, Oensingen, Switzerland) with sandblasted surfaces	10 Immediate implant placement (5 occ, 5 non-occ) 30 late placement	Implant preparation and single immediate acrylic crowns, occ: immediately occlusally loaded non-occ: non-occlusally loaded ceramic crowns after 4–5 months	Overall 87.5 Occ: 85 Non-occ: 90	NR	Occ 0.9 ± 0.48 Non-Occ 0.7 ± 0.59
5	Kohal 2012	Prospective case series	12 months	65	66	One-piece	ZiUnite zirconia implants (Nobel Biocare, Gothenburg, Sweden) a machined collar with a roughened transmucosal part, a tapered and roughened endosseous part using a sintering-on technique	Immediate implant placement or in healed sites using flapless, punch or flap techniques	Implant preparation and immediate temporization, then single crowns after: mand: minimum 6 weeks max: minimum 14 weeks	95.4	Success criteria (Ostman et al. 2007, 2008) grade I: 66 grade II: 86	1.31
6	Kohal 2013	Prospective case series	12 months	28	56	One-piece	ZiUnite zirconia implants (Nobel Biocare, Gothenburg, Sweden) a machined collar with a roughened transmucosal part, a tapered and roughened endosseous part using a sintering-on technique	Immediate implant placement or in healed sites using flapless, punch or flap techniques bone augmentation without membranes when needed	Implant preparation and immediate temporization, then FDPs after: mand: minimum 6 weeks max: minimum 14 weeks	98.2	Success criteria (Ostman et al. 2007, 2008) grade I: 60 grade II: 72	1.95
7	Borgonovo 2013	Prospective case series	48 months	13 (10 at follow-up)	35 (28 at follow-up)	One-piece	WhiteSKY zirconia implants (Bredent, Senden, Germany) sandblasted endosseous surface	Late implant placement with full thickness flap reflection regenerative procedures used when required	Immediate implant abutment preparation and temporary restorations final CAD/CAM all ceramic zirconia SC or FDP 6 months after	100	100	1.63
8	Payer 2013	Prospective case series	24 months	20	20	One-piece	WhiteSKY zirconia implants (Bredent, Senden, Germany) sandblasted endosseous surface	Late implant placement with full thickness flap reflection no bone augmentation	Immediate CAD/CAM provisional adhesively cemented restoration (out of occlusion) all ceramic SC after 4 months of healing (provisional ground and used as a cap under the final restoration)	95	95	1.29
9	Osman 2014	RCT	12 months	24 (19 at follow-up) Ti 12 (8 at follow-up) Zr 12 (11 at follow-up)	129, Ti 56 Zr 73	One-piece	Southern Implants (Irene, South Africa) with tapered threaded implant body, a transmucosal cylindrical collar, and a ball abutment Zr: One-piece Zr implants with acid etched surfaces	Late implant placement with full thickness flap reflection except for palatal implants	Implant-supported overdentures 3–4 months after implant placement maxilla: 2 implants in the premolar regions, 1 off-center and 1 palatal implant mandible: 2 distal implants in the molar	Overall Zr 71.2 overall Ti 82.1 mand. Zr 90.9 mand. Ti 95.8 max. Zr 55	NR	Zr 0.42 ± 0.40 Ti 0.18 ± 0.47
10	Payer 2015	RCT	24 months	22	31, Zr 16 Ti 15	Two-piece	Ziterion Vario z, yttria-stablized zirconia implants Ziterion Vario t, titanium implants (Ziterion GmbH, Uffenheim, Germany)	Minimum 6 months healing period	Abutments cemented at 2nd stage surgery under rubber dam isolation 4–6 months after implant placement all-ceramic single crowns	Zr 93.3 Ti 100	Zr 93.3 Ti 100	Zr 1.48 ± 1.05 Ti 1.43 ± 0.67
11	Brull 2014	Retrospective	Mean 18 months	74	121, two-piece 66 one-piece 55	One and two-piece	Individually designed implants milled from round, isostatically pressed yttria-stabilized and cerium co-stabilized zirconia blanks, air particle abraded then sintered	Immediate or late placement	Mean healing period 4.6 ± 3–17 months SC: 82.6 % FDPs: 17.4 %	96.5	NR	0.1 ± 0.6
12	Cionca 2015	Prospective case series	Mean 588 ± 174 days	32	49	Two-piece	Zeramex T implants with sandblasted acid-etched surfaces	Late placement in healed sockets	Mean healing period 193 ± 79 days, cemented all ceramic SC	87	NR	NR
13	Spies 2015	Prospective	12 months	27	27	One-piece	Alumina toughened zirconium dioxide ATZ (Ziraldent FR1, Metoxit AG, Thayngen, Switzerland) Zircapore surface (sandblasted with a ceramic slurry coating) Tapered, self-tapping implants with reduced diameter at the transition zone from soft to hard tissues	Late placement in healed sockets	SC immediate provisional restoration then CAD/CAM all ceramic crowns in: mand: 6 weeks max:14 weeks	88.9	Success criteria (Ostman et al. 2007, 2008) grade I: 91.7 grade II: 100	0.77
14	Roehling 2015	Retrospective	Mean 5.94 ± 0.09 years	71	161, 3.25 mm diameter 51 (31.7 %) 4.0 mm diameter 82 (50.9 %) 5.0 mm diameter 28 (17.4 %)	One-piece	Z-Look 3 (Z-Systems GmbH, Kiel, Germany) with sandblasted surfaces	At least 6 weeks post-extra	At least 3 months healing period (implants immediately protected from premature loading) SC 69 % FDP 19.3 % removable hybrid dentures 2.5 %	Overall 77.3 3.25 mm implants 58.5 4.0 mm implants 88.9 5.0 mm implants 78.6	Overall 77.6 3.25 mm implants 58.8 4.0 mm implants 89 5.0 mm implants 78.6	0.97 ± 0.07

Success grade I (Ostman et al. 2007): implants with no clinical or radiographic signs of pathology, showing ≤2 mm bone resorption at the 1-year follow-up. Success grade II (Ostman et al. 2007): implants with no clinical or radiographic signs of pathology, showing ≤3 mm bone resorption at the 1-year follow-up

*RCT* randomized controlled clinical trial, *MBL* marginal bone loss, *NR* not reported, *SC* single crown, *FDP* fixed dental prosthesis, *Mand* mandible, *Max* maxilla, *CAD/CAM* computer-aided design/computer-aided manufacturing, *Zr* zirconia implants, *Ti* titanium implants, *UC* uncoated implant surfaces, *C* coated implant surfaces with Na_2_O–K_2_O–MgO–Al_2_O_3_–CaO–SiO_2_–P_2_O_5_–F, *ICE* acid etched implant surfaces

### Excluded studies

Out of the 39 publications reviewed in details, 25 were excluded from the final analysis (Table 2). The main reasons for exclusion were the following:

**Table 2:** Excluded studies and reasons for exclusion

	Author, year	Reason for exclusion
1	Kohal 2004	Sample size
2	Oliva 2007	The same group of patients included in Oliva 2010
3	Oliva 2008	Sample size
4	Oliva 2008, 2	Sample size
5	Pirker & Kocher 2008	Sample size
6	Oliva 2010, 2	Sample size
7	Walker 2010	Sample size
8	Borgonovo 2010	The same group of patients included in Borgonovo 2014
9	Arnetzl 2010	Sample size
10	Nevins 2011	Sample size
11	Pirker 2011	Sample size
12	Borgonovo 2011	The same group of patients included in Borgonovo 2014
13	Borgonovo 2012	The same group of patients included in Borgonovo 2014
14	Pirker & Kocher 2012	Sample size
15	Oliva 2012	Titanium implants with zirconia superstructures
16	Borgonovo 2013	The same group of patients included in Borgonovo 2014
17	Borgonovo 2013, 2	The same group of patients included in Borgonovo 2014
18	Osman 2013	Sample size
19	Gahlert 2013	The same group of patients included in Roehling 2015
20	Aydin 2013	Sample size
21	Nair 2013	Sample size
22	Bankoglu 2014	Sample size
23	Spies 2014	The same group of patients included in Kohal 2012, 2013, but this study evaluated the survival of prosthetic superstructures
24	Siddiqi 2015	The same group of patients included in Osman 2014
25	Gahlert 2015	Functional loading period less than 1 year

- Sample size.
- Observation period of less than 1 year after loading.
- Unclear surgical and/or prosthetic protocol.
- Studies examining the same group of patients.

### Quality assessment

Table 3 shows the list of studies detailing the criteria used for quality assessment. One study [22] was considered highly biased due to unavailability of details on neither clinical nor radiologic examinations, lack of adjustment for different surgical protocols, and lack of statistical analysis. Six articles [20, 21, 23–26] were considered to have a moderate degree of bias, while the remaining seven [27–33] studies had a low degree of bias.

**Table 3:** Quality assessment of the included studies

	Study ID	Design	Evidence level^a^	Detailed clinical exam	Rx: quality and interpretation	Adjustment for different surgical and loading protocols	Completeness of follow-up	Statistical analysis	Industry funding	Risk of bias
1	Blaschke 2006	Prospective	III	No	No	No	Yes	No	Yes	High
2	Pirker and Kocher 2009	Prospective	III	Yes	No	No	Yes	Yes	Unclear	Moderate
3	Cannizzaro 2010	RCT	Ib	No	Yes	Yes	Yes	Yes	Yes	Low
4	Oliva 2010	Prospective	III	No	No	Yes	Yes	Yes	Unclear	Moderate
5	Kohal 2012	Prospective case series	III	Yes	Yes	No	Yes	Yes	Yes	Moderate
6	Kohal 2013	Prospective case series	III	Yes	Yes	No	Yes	Yes	Yes	Moderate
7	Borgonovo 2013	Prospective	III	Yes	Yes	Unclear	Yes	Yes	No	Low
8	Payer 2013	Prospective case series	III	Yes	Yes	Yes	Yes	Yes	Yes	Low
9	Osman 2013	RCT	Ib	No	Yes	No	Yes	Yes	Unclear	Moderate
10	Payer 2015	RCT	Ib	Yes	Yes	Yes	Yes	Yes	Yes	Low
11	Cionca 2015	Prospective case series	III	Yes	No	Yes	Yes	Yes	Yes	Low
12	Brull 2014	Retrospective	III	Yes	Yes	No	Yes	Yes	Yes	Moderate
13	Spies 2015	Prospective	III	Yes	Yes	Yes	Yes	Yes	Yes	Low
14	Roehling 2015	Retrospective	III	Yes	Yes	Yes	Yes	Yes	Yes	Low

^a^According to the definitions of types of evidence originating from the US Agency for Health Care Policy and Research (1993)

### Assessment of heterogeneity and meta-analysis

Preliminary examination of the survival of implants at 1 year for the selected studies revealed considerable heterogeneity, (*I*^2^ = 79.3 %, tau-squared = 0.698, *p* < 0.0001). Information on each study’s characteristics are detailed in Table 1.

### Description of included studies

#### One-piece implants

Eleven studies evaluated one-piece implant systems and one included both one- and two-piece implants. Of these, five investigations examined both immediate and late implant placement, and one did not report the timing of implant surgery.

In the first study [22], 34 patients with 66 zirconia implants were monitored over a period of 2 to 5 years. The fixtures were either splinted or protected with special prostheses during a healing period of 4 to 6 months. However, details regarding timing, surgical protocol, clinical and radiographic examinations were not provided. The authors reported good osseointegration related to 98 % of the implants 1 to 2 years following implantation. Only one implant was fractured due to external trauma, and thereby extracted and subjected to histological evaluation. This revealed direct BIC with neither a fibrous layer nor signs of a foreign body reaction.

Another study [23] evaluated immediate, non-submerged, root-analogue zirconia implants with two different surfaces for single-rooted tooth replacement. Six patients received root-identical replicas with sandblasted implant surfaces, while 12 patients received modified implants with added interdental macro-retention and a slightly reduced bucco-lingual dimension. Implants were inserted 1 to 8 days after tooth extraction by tapping, which resulted in immediate limited functional loading. All six implants in the first group failed prior to prosthetic restoration. The 12 patients in the second group received single composite crowns after a healing period of 3 to 5 months. The overall survival rate of the modified implants was 92 % after 1–33 months of function. The authors reported excellent esthetic and functional results with minimal bone resorption and soft tissue recession.

A third study [24] evaluated the 5-year success rate of 831 zirconia implants with three different surfaces: uncoated (UC, *n* = 249), coated (C, *n* = 249), and acid-etched (ICE, *n* = 333). The UC implants were roughened by mechanical grinding, while the C implants were roughened and coated with a bioactive ceramic coating composed of Na_2_O–K_2_O–MgO–Al_2_O_3_–CaO–SiO_2_–P_2_O_5_–F, then sintered. This investigation included immediate as well as late implant placement, with or without simultaneous bone augmentation, as well as one- or two-stage sinus lift. Three hundred seventy-eight patients, with a mean follow-up period of 3.4 years, were examined. The overall 5-year success rate was 95 %, with ICE implants showing significantly higher success rate compared to both the UC and C ones.

A multi-center randomized controlled clinical trial [27] further compared 20 single non-occlusally loaded zirconia implants with 20 occlusally loaded implants after 1 year of function. Five implants in each group were placed in fresh extraction sockets. Overall, five implants (12.5 %) failed early; four of which were immediately placed after tooth extraction, and three were occlusally loaded. Both occlusal and non-occlusal implants showed significant marginal bone loss after 1 year of loading, but the difference was not statistically significant between groups. The authors concluded that there was an association between immediate implants and implant failure.

Another group investigated one-piece zirconia implants for single-tooth replacement or 3-unit fixed dental prosthesis (FDP) in two consecutive publications. The first [20] included 65 patients treated with 66 single one-stage implants and immediate temporization. Five implants (9 %) were placed in fresh extraction sites, 19 (27 %) were placed in healed sites using a flapless technique, and 42 (64 %) were placed after flap elevation. Three implants failed early prior to prosthetic restoration leading to a cumulative survival rate of 95.4 % after 1 year. A mean marginal bone loss (MBL) of 1.31 mm was reported, with 19 implants (34 %) losing at least 2 mm of bone, and 8 (14 %) losing more than 3 mm of marginal bone. Yet, stable and healthy peri-implant soft tissue conditions were noted at the 1 year follow-up. Regardless, the authors could not recommend the use of the tested implant system in clinical practice.

The second publication [21] evaluated the 1 year results of 3-unit FDPs in 28 patients with 56 implants. Five implants (9 %) were immediately placed (2 after flap elevation); 51 implants were placed in healed sites (5 using the punch technique and 2 flapless). Only one implant belonging to the immediately placed group failed prior to prosthetic reconstruction, resulting in a survival rate of 98.2 % after 1 year. The mean MBL was 1.95 mm after 1 year. However, 10 patients (40 %) showed at least 2 mm of MBL, while 7 (28 %) lost more than 3 mm, and 3 (12 %) lost more than 4 mm of marginal bone. A correlation was found between MBL and the flap design. Implants placed using a flapless approach or the punch technique showed significantly more MBL than those placed after flap elevation. Finally, due to the high frequency and increase in radiographic bone loss around the tested implants, the authors concluded that this one-piece zirconia implant system might perform inferiorly to conventional titanium implant systems and to other zirconia implants in terms of MBL.

A 4-year clinical and radiographic study [28] evaluated 13 patients with 35 zirconia implants placed in healed sites. Twenty implants were used for multiple teeth replacement while the rest replaced single teeth. However, only 10 patients with 28 implants were available for the final examination. Success and survival rates were calculated at 100 % after 48 months. The mean MBL was 1.631 mm at the end of the follow-up period, with maxillary implants showing significantly higher MBL during the first year of loading when compared to mandibular ones. In contrast, no differences were found between implants restored with single crowns (SC) or FDPs in terms of MBL. Finally, the authors stated that minimal plaque accumulation, no bleeding, and a probing depth (PD) of 3.19 mm could be expected around zirconia implant-supported restorations.

Another prospective case series [29] evaluated the outcomes of 20 single-piece, immediately provisionalized, zirconia implants placed in single-tooth gaps after a period of 2 years. The results showed 95 % survival and success rates with a mean MBL of 1.29 mm at the end of the observation period. Clinical parameters showed healthy soft tissue conditions and an improved, but not significant, pink esthetic score [34] after 24 months. Regardless of such promising results, the authors refrained from drawing final conclusions or clinical recommendations.

One-piece zirconia implants were also evaluated as abutments supporting overdentures, in comparison with titanium implants of similar design [26]. This randomized controlled clinical trial included 24 edentulous patients with 129 implants randomly divided into two groups: the zirconia test group and the titanium control group. Each participant received four maxillary implants distributed in a diamond-shaped quad design (one mid-palatal and three anterior crestal), and three mandibular implants with a tripod design (one mid-symphyseal and two bilateral distal). There was no significant difference in the survival rate between the groups, but the overall survival rate of 71.2 % was considered low in comparison with other zirconia implant trials. Regarding mandibular implants, the survival rate of the titanium group was 95.8 % compared to 90.9 % for zirconia implants. The maxillary implants’ survival rates were 71.9 and 55 % for the titanium and zirconia implants, respectively. Statistical analysis showed a significantly higher risk of failure for maxillary implants. The mean MBL was 0.18 mm for titanium and 0.42 mm for zirconia implants for both jaws combined. In contrast to implants placed in the upper arch, significantly higher MBL was found around zirconia implants placed in the mandible when compared to the titanium group. Moreover, three zirconia implants fractured, two of which were located in the maxillary jaw, resulting in the recommendation of at least four wider diameter fixtures for maxillary overdenture support when using zirconia implants. Further modifications of implant design to improve biomechanics integrity were also recommended. Finally, the authors advised for caution before recommending the use of single-piece zirconia implants for overdenture support.

A more recent prospective investigation [33] was conducted to determine the clinical and radiographic outcomes of one-piece alumina-toughened zirconia implants for single-tooth replacement in 27 patients. Three implants were lost early prior to prosthetic reconstruction. Hence, 24 patients were seen at the 1-year follow-up, resulting in a survival rate of 88.9 %. The mean MBL was 0.77 mm at follow-up, with only two implants (8.3 %) losing at least 2 mm of bone. Probing depth (PD) and calculated attachment level (CAL) increased while recession remained stable during the observation period. Mean bleeding (mBI) and plaque (mPI) indexes showed no statistically significant changes within the first year. The authors finally concluded that the tested implant system showed promising short-term results and seemed to be a candidate for clinical use.

Another recent study [32] examined zirconia one-piece implants after up to 7 years of loading. A total of 71 patients with 161 implants and a mean follow-up period of 5.94 years were included in this analysis. The overall survival rate was 77.3 %. Implants with reduced diameter (3.25 mm) showed the lowest survival rate at 58.5 % in comparison with implants of 4.0 and 5.0 mm diameter at 88.9 and 78.6 %, respectively. Fourteen implants were lost prior to prosthetic reconstruction, 4 failed late, and 18 implants were fractured at the coronal part of the sandblasted implant body. The authors concluded that the first-generation zirconia implants investigated showed low overall survival and success rates. They also noted that non-fractured failures were not associated with peri-implant infections.

#### Two-piece implants

Only two clinical studies evaluating two-piece zirconia implants were included in the current analysis. The first was a prospective study [30] that included 32 patients treated with 49 implants supporting single crowns. The cumulative survival rate was 87 % after 1 year of loading. All failures were due to aseptic loosening. Furthermore, the authors reported neither soft tissue complications nor MBL exceeding 2 mm at the end of the observation period.

The second study was a randomized clinical trial [31] that evaluated 16 zirconia implants in comparison with 15 titanium implants of identical shape in 22 patients. After up to 2 years of loading, the survival rate was 93.3 and 100 % for zirconia and titanium implants, respectively. The mean MBL was 1.48 mm for zirconia and 1.43 mm for titanium. The authors further concluded that zirconia implants’ survival rate and clinical outcomes showed no significant differences in comparison with titanium implants.

One study [25] retrospectively analyzed the clinical performance of both one and two-piece implants in 74 participants over a period of 3 years. A hundred twenty-one implants (55 one-piece and 66 two-piece) were evaluated after a mean observation period of 18 months. The cumulative survival rate of 96.5 % was calculated after 3 years, and the surviving implants showed healthy mucosal conditions with significantly lower bleeding on probing and PD around implants when compared to teeth.

### Implant survival

**Fig. 2:**
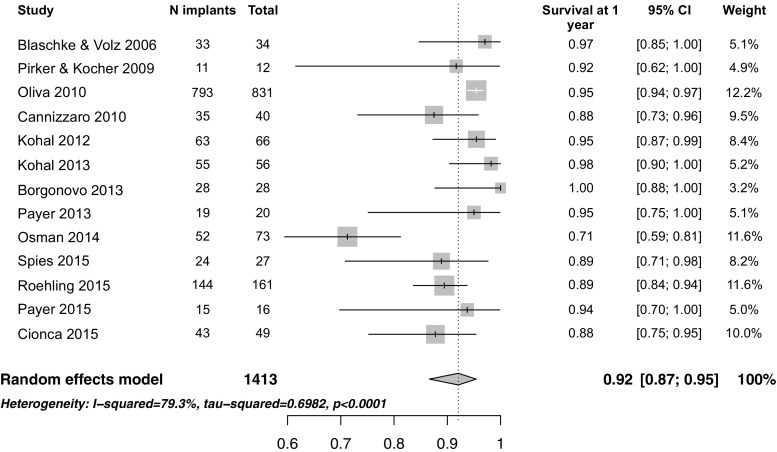
Forest plot for the survival of zirconia implants after 1 year of function when all selected studies were included except Brull et al. [25]

All but two studies reported cumulative survival rates after at least 1 year of loading. Cannizzaro et al. reported failure rates, which were used for calculation of the survival rate after requesting the author’s permission [27]. On the other hand, the 1-year survival rate could not be extrapolated for the study conducted by Bull et al. who reported the 3-year survival rate of both one- and two-piece implants [25]. Therefore, this study was excluded from the quantitative analysis. Only one study reported survival of one-piece implants after 4 years [28], while two others reported the cumulative survival rates after 5 [24] and 7 years [32]. Yet, the meta-analysis was limited to survival of implants at 1 year due to the limited observation period in most studies. The overall survival rate of zirconia one- and two-piece implants was 92 % (95 % CI 87–95) after 1 year of function (Fig. 2).

**Fig. 3:**
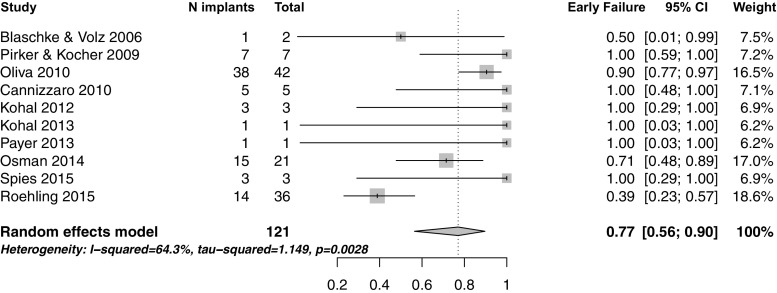
Forest plot for the early failure of zirconia one-piece implants where only the studies evaluating one-piece implants were included with the exception of Borgonovo et al. [28] and Brull et al. [25]

Table 4 shows the prevalence of early and late failures across the studies, and Fig. 3 shows the forest plot for the early failures of one-piece zirconia implants. However, the meta-analysis was done on one-piece implants excluding Borgonovo et al. who presented data on 28 surviving implants after 4 years of function, and hence, no failures were reported in this publication [28]. Brull et al. [25] was also excluded because they examined both one- and two-piece implants without distinction. Early failure of one-piece zirconia implants ranged between 1.8 [21] and 100 % [23], with the overall early failure rate calculated at 77 % (95 % CI 56–90). On the other hand, only two [30, 31] out of the three studies evaluating two-piece zirconia implants clearly reported failure rates. Cionca et al. reported a failure rate of 12.2 % with only one early failure (2 %) compared to five (10.2 %) late failures [30]. Payer et al. showed a 6.3 % failure rate with only one implant failing after prosthetic rehabilitation [31]. In contrast, Brull et al. only reported the loss of three implants (one early failure, one late failure, and one fractured implant) without details on the implant design [25]. Thus, meta-analysis could not be performed on the early failure of two-piece implants.

**Table 4:** Failure rate and the prevalence of early failure, late failure, and implant fracture in the selected studies

Author, year	Observation period	*N* of implants	Calculated failure rate (%)	*N* of early failures (%)	*N* of late failures (%)	*N* of fractured implants (%)
One-piece implants						
Blaschke and Volz 2006	2–5 years	34	2	1 (2.9)	0	1 (2.9)
Pirker and Kocher 2009	Mean 18 months	Group A: 6	Group A: 100	Group A: 6	(100)	0
		Group B: 12	Group B: 8	Group B: 1 (8.3)	0	0
Oliva 2010	Mean 40.8 months	831	5.05	38 (4.6)	4 (0.5)	0
Cannizzaro 2010	12 months	40	12.5	5 (12.5) 3 occlusal, 2 non-occlusal	0	0
Kohal 2012	12 months	66	4.6	3 (4.6)	0	0
Kohal 2013	12 months	56	1.8	1 (1.8)	0	0
Borgonovo 2013	48 months	28	0	0	0	0
Payer 2013	24 months	20	5	1 (5)	0	0
Osman 2014	12 months	73	28.7	15 (20.6)	3 (4.1)	3 (4.1)
Spies 2015	12 months	27	11.1	3 (11.1)	0	0
Roehling 2015	Mean 5.94 years	161	22.4	14 (8.7)	4 (2.5)	18 (11.2)
Two-piece implants						
Payer 2015	24 months	16	6.3	0	1 (6.3)	0
Cionca 2015	Mean 588 days	49	12.2	1 (2)	5 (10.2)	0
One and two-piece implants						
Brull 2014	Mean 18 months	121	2.5	1 (0.8)	1 (0.8)	1 (0.8)

## Discussion

**Fig. 4:**
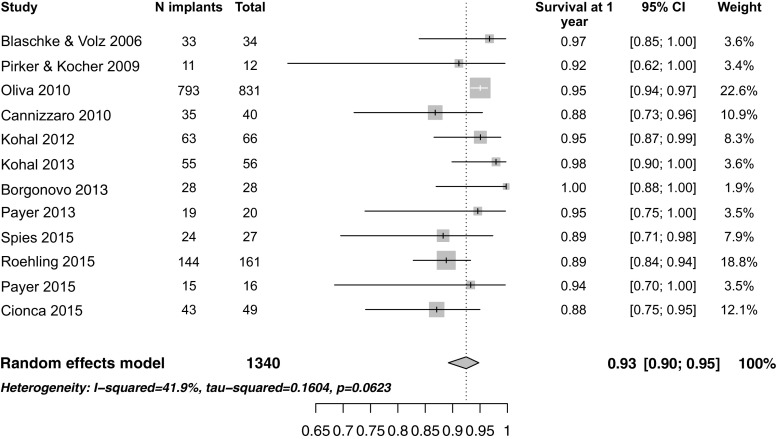
Forest plot for the survival of zirconia implants after 1 year of function excluding Osman et al. [26] and Brull et al. [25]

This systematic review and meta-analysis focused on clinical studies evaluating the survival rate of zirconia implants after 1 year of function. In contrast to previous reviews, which either evaluated animal studies or were only narrative, only clinical studies with an observation period of at least 1 year were included in this analysis. The overall survival rate of zirconia implants was 92 % (95 % CI 87–95) after 1 year of function, with significant heterogeneity between the studies (*I*^2^ = 79.3%, tau-squared = 0.698, p <0.0001). In comparison, the overall survival rates of titanium implants supporting single crowns (SC) was 97.2 % at 5 years and 95.2 % at 10 years [35]. While the survival rates of titanium implants supporting fixed dental prosthesis (FDP) was 97.2 and 93.1 % after 5 and 10 years, respectively [36]. Yet, when the prosthetic design is taken into consideration, thereby excluding Osman et al. [26] due to their unconventional prosthetic design, the heterogeneity between the studies decreased to an insignificant level (*I*^2^ = 41.9 %, tau-squared = 0.16, *p* = 0.06). Moreover, the cumulative survival rate for zirconia implants with fixed reconstructions increased to 93 % (95 % CI 90–95) after 1 year of function (Fig. 4). Osman et al. compared both alveolar and palatal zirconia implants to titanium ones as abutments for overdentures. The overall survival rate was 71.2 % for zirconia and 82.1 % for titanium implants. This generally low survival was attributed to the implants’ one-piece design and their moderately rough surface being in contact with the mucosa, as well as the flapless surgical protocol, the unconventional distribution of the implants, and the immediate loading protocol. Furthermore, their results were affected by the high failure rate of mid-palatal implants (42.1 %), which was believed to be due to either direct trauma from tooth brushing or parafunctional tongue activity.

The survival rates for zirconia implant-supported fixed reconstructions ranged from 87 to 100 %. Yet, Cannizzaro et al. [27] who reported a survival of 87.5 % at 1 year evaluated different loading protocols (immediate occlusal or non-occlusal), and 10 out of the 40 implants examined were inserted into fresh extraction sockets. This could account for the lower survival rate of their implants. Moreover, Spies et al. [33], who reported a survival of 88.9 % at 1 year, examined one-piece alumina-toughened zirconia implants. The three implants that failed to osseointegrate were among the first inserted, and their early failure was attributed to the immediate temporization required for one-piece implants and the initial healing period that is highly dependant on the patient’s good compliance as well as the clinician’s practical values. Cionca et al. [30] further reported a survival rate of 87 % for a two-piece implant system with an acid-etched sandblasted surface. In this study, only one implant failed to osseointegrate while five others were lost 1 to 10 months after loading due to aseptic loosening. The implants’ experimental design and the type of surface treatment used could have contributed to the lower survival rate when compared to other studies.

When the failure patterns of zirconia implants were examined, one-piece zirconia implants demonstrated a higher tendency towards early failure (Table 4 and Fig. 3), with the overall early failure rate calculated at 77 % (95 % CI 56–90). However, the meta-analysis included a study conducted by Pirker and Kocher which included two types of implants. All six implants in the first group failed early, while only one out of the 12 implants in the second group was lost. Still, the seven reported failures were included in the meta-analysis of the early failure which could have confounded the results [23]. Furthermore, only one study [32] reported a high fracture rate of 11.2 % during a mean observation period of 5.9 years, while three others [22, 25, 26] reported low implant fracture rates ranging between 0.8 and 4 %. Moreover, the single fracture reported by Blaschke et al. was due to external trauma [22]. On the other hand, the two studies examining two-piece implants [30, 31] reported a higher percentage of late compared to early failure, and no fractured implants (Table 4). Yet, the significant heterogeneity of the studies and the scarcity of data on two-piece implants hindered statistical analysis.

The results of this analysis should be interpreted with caution for several reasons. First, the majority of the analyzed studies were case reports with limited sample sizes and short-term follow-up. Second, the selected studies examined zirconia implants with considerable variability in implant design, surface characteristics, surgical protocols, and prosthetic superstructures. Six studies reported on outcomes after immediate implant placement [20, 21, 23–25, 27], which has been proven to have significantly lower survival rates for titanium implants [37]. Furthermore, the heterogeneity between studies regarding the type of implant surface treatment, which significantly affects osseointegration [38–40], could account for the differences in survival rates. Out of the 14 studies included in this investigation, only Oliva et al. compared implants with different surface modifications. They established that acid-etched zirconia implants had significantly higher survival rates (97.6 %) when compared to the simply roughened uncoated or coated implants, at 92.77 and 93.57 %, respectively [24]. Comparison of a certain type of surface treatment across studies could not be done due to the high variability between studies in that respect. However, since none of the studies utilized machined implants, and since multiple studies showed better osseointegration of roughened zirconia implants regardless of the surface treatment used [13, 38, 39, 41–43], pooling the data was considered appropriate. However, combining the data from one- and two-piece implant systems was still considered one of the downsides of this analysis. This was unavoidable due to the scarcity of reports on two-piece zirconia implants. Also, limitations of one-piece implant systems should be taken into consideration. The sparse options for abutment angulation present a major difficulty that could compromise the surgical positioning of the implant. Furthermore, preparation of sub-optimally positioned implants should be avoided due to its adverse effects on the material’s physical properties, as well as the lack of data on the long-term stability afterwards. Single-piece implants also require a load-free healing period, which could be challenging due to the inevitable immediate forces directed at the supra-mucosal part during mastication or with tongue movement [6, 8, 12]. A review [6] evaluating one-piece zirconia implants showed survival rates ranging between 74 % and 98 % after 12–56 months, with success rates varying between 79.6 % and 91.6 % after 6–12 months of function. However, a small number of studies with limited observation periods were available for this analysis. Two-piece zirconia implants were introduced to overcome complications associated with one-piece systems, but their development has been hindered by the material’s physical properties, and only few clinical studies evaluated the outcomes of zirconia two-piece implants [25, 30, 31, 44]. This sheds light on the urgent need for further studies examining such implants.

An additional drawback to this review was the type of fixed reconstructions evaluated, as all selected studies examined cemented SCs or FDPs. This was attributed to the lack of screw-retained zirconia implant-supported restorations due to the material’s physical limitations. However, excess cement presents a frequent and major complication that has been proven to provoke an inflammatory reaction around titanium implants [45, 46]. Yet, incidence of peri-implantitis has never been reported in conjunction with zirconia implants. It remains to be determined whether this is due to the higher biocompatibility of zirconia ceramics or if it is merely due to the lack of studies on the subject. Finally, this analysis did not address the high MBL associated with zirconia implants, which could be the focus of a future review.

## Conclusions

In spite of the unavailability of sufficient long-term evidence to justify using zirconia oral implants, zirconia ceramics could potentially be the alternative to titanium for a non-metallic implant solution. However, further clinical studies are required to establish long-term results, and to determine the risk of technical and biological complications. Finally, additional RCTs examining two-piece zirconia implant systems are required to assess their survival and success rates in comparison with titanium and one-piece zirconia implants.
